# 非M_3_型急性髓系白血病合并弥散性血管内凝血的危险因素与预后分析

**DOI:** 10.3760/cma.j.cn121090-20251125-00548

**Published:** 2026-06

**Authors:** 信琦 韩苗, 晓燕 徐, 虹 王, 德沛 吴, 悦 韩

**Affiliations:** 苏州大学附属第一医院，江苏省血液研究所，国家卫生健康委员会血栓及止血重点研究室，国家血液系统疾病临床医学研究中心，苏州 215006 The First Affiliated Hospital of Soochow University, Jiangsu Institute of Hematology, Key Laboratory of Thrombosis and Hemostasis of Ministry of Health, National Clinical Research Center for Hematologic Diseases, Suzhou 215006, China

## Abstract

本研究回顾性分析2016至2024年苏州大学附属第一医院407例初治非M_3_型急性髓系白血病（AML）患者，依据ISTH-2018弥散性血管内凝血（DIC）评分分为DIC组109例和非DIC组298例，比较两组的临床特征及生存差异。结果显示，DIC组外周血白细胞计数、骨髓原始细胞比例、丙氨酸转氨酶、天冬氨酸转氨酶及乳酸脱氢酶水平均显著高于非DIC组（均*P*<0.001）；分子学分析提示，NPM1、FLT3-ITD及SETD2突变在DIC组中显著富集（均*P*<0.05）。Logistic多因素分析表明，高白细胞计数、原始细胞比例升高、乳酸脱氢酶升高及NPM1突变是DIC发生的独立危险因素（均*P*<0.05）。临床结局分析显示，DIC组早期重度出血发生率更高（*P*<0.001），首疗程诱导化疗完全缓解率更低（*P*<0.05）；在总人群中，尤其在ELN2022危险分层高危亚组中，DIC组总生存明显差于非DIC组（*P*<0.05）。Cox多因素分析进一步证实，合并DIC、早期重度出血及高龄是影响非M_3_型AML患者总生存的独立危险因素（均*P*<0.05）。上述结果提示，非M_3_型AML合并DIC与较高肿瘤负荷及特定分子学异常密切相关，并伴随更高的早期出血风险和更差的生存结局，提示DIC可能代表一类更高危的临床特征，需加强早期监测与分层管理。

## Abstract (en)

This study retrospectively analyzed 407 newly diagnosed patients with non-M_3_ acute myeloid leukemia（AML）treated at the First Affiliated Hospital of Soochow University between 2016 and 2024. Based on the ISTH-2018 DIC score, patients were classified into a DIC group（*n*＝109）and a non-DIC group（*n*＝298）, and their clinical characteristics and survival were compared. The results showed that white blood cell count, bone marrow blast percentage, alanine aminotransferase, aspartate aminotransferase, and lactate dehydrogenase levels were significantly higher in the DIC group than in the non-DIC group（all *P*<0.001）. Molecular analysis indicated that NPM1, FLT3-ITD, and SETD2 mutations were significantly enriched in the DIC group（all *P*<0.05）. Multivariable logistic regression showed that a high white blood cell count, a high blast percentage, an elevated lactate dehydrogenase level, and NPM1 mutation were independent risk factors for DIC（all *P*<0.05）. Clinical outcome analysis showed that the DIC group had a higher incidence of early severe bleeding（*P*<0.001）and a lower complete remission rate after first induction chemotherapy（*P*<0.05）. In the overall population, especially in the European LeukemiaNet（ELN）2022 high-risk subgroup, overall survival（OS）was significantly worse in the DIC group than in the non-DIC group（*P*<0.05）. Multivariable Cox regression further confirmed that concomitant DIC, early severe bleeding, and older age were independent risk factors for OS in patients with non-M_3_ AML（all *P*<0.05）. These findings suggest that DIC in non-M_3_ AML is closely associated with a high tumor burden and specific molecular abnormalities and is accompanied by a higher risk of early bleeding and poorer survival outcomes. DIC may therefore represent a higher-risk clinical feature requiring enhanced early monitoring and risk-stratified management.

弥散性血管内凝血（DIC）是一种获得性的、危及生命的血管性疾病[1]。急性白血病患者常合并严重凝血异常，临床表现包括皮肤黏膜出血及DIC引起的不同程度出血[2]。严重出血是急性早幼粒细胞白血病（APL，M_3_）患者早期死亡的主要原因[3]。然而，部分非M_3_型急性髓系白血病（AML）患者在初诊时也常伴有凝血功能异常，疾病过程中存在一系列凝血与纤溶失衡[4]。非M_3_型AML患者的DIC发生率在8.5％～25％不等[5]，伴DIC的患者合并系统并发症比例增加，早期死亡风险显著升高[6]。APL合并DIC的病理生理机制及治疗管理已被广泛研究，但是非M_3_型AML合并DIC的报道相对有限，其高危因素及发生机制尚不清楚[7]。本研究通过分析非M_3_型AML相关DIC的危险因素及预后，为探寻DIC发生的机制提供依据，为早期干预及临床决策提供支持。

## 病例与方法

1. 病例资料：回顾性分析2016年10月至2024年8月在苏州大学附属第一医院首次诊断为非M_3_型AML的患者，共407例，诊断均遵循2016版WHO白血病分类[8]。纳入对象需具备完整的骨髓细胞形态学及分子遗传学资料，包括骨髓细胞比例、融合基因、基因突变和染色体核型结果，同时具备完整的临床资料以支持DIC评分。排除既往已接受诱导化疗后转入本中心者、APL患者及继发于骨髓增生异常肿瘤的AML患者。

2. 临床资料收集：包括基本信息、血细胞分析、凝血系列、肝肾功能、乳酸脱氢酶（LDH）、骨髓涂片原始细胞百分比、染色体核型、融合基因以及二代测序（NGS）结果。所有患者均在入院24 h内完成外周血及骨髓标本的采集。遗传学及分子学指标危险度分级根据欧洲白血病网（ELN）2022指南标准[9]。DIC评分基于入院24 h内、治疗开始前获得的血小板计数及凝血指标，遵循2018-ISTH DIC评分[10]，将患者分为DIC组（109例）和非DIC组（298例）。

3. 治疗方案：传统化疗方案：包括IA（去甲氧柔红霉素+阿糖胞苷）、HA（高三尖杉酯碱+阿糖胞苷）、HAA（高三尖杉酯碱+阿糖胞苷+阿克拉霉素）±G-CSF±地西他滨方案；包含维奈克拉方案：维奈克拉+去甲基化药物（阿扎胞苷或地西他滨），维奈克拉+阿糖胞苷+去甲氧柔红霉素/高三尖杉酯碱方案。在本队列中，年龄较轻且体能状态良好的患者接受强化疗方案（如IA、HAAG等），而高龄或体能状态较差的患者则主要采用低强度治疗方案，包括去甲基化药物联合维奈克拉等方案。化疗后骨髓原始细胞比例<5％，外周血未见原始细胞，无髓外病变，外周血中性粒细胞≥1.0×10^9^/L且PLT≥100×10^9^/L定义为完全缓解（CR）。

4. 早期出血事件评价：出血事件根据WHO量表确定出血等级[11]，Ⅲ、Ⅳ度被定义为重度出血。患者自确诊至首次化疗后60 d内发生的出血事件被定义为早期出血事件[12]。

5. 随访：随访截止时间为2025年9月1日，随访资料来源于门诊病历、住院病历、电话随访记录等，总人群的中位随访时间为39（1～104）个月，失访患者58例（14.2％），总生存（OS）时间定义为首次确诊至死亡或随访截止的时间。

6. 统计学处理：采用SPSS 27.0软件进行统计分析。所有连续变量均符合偏态分布，以中位数（范围）表示，采用Mann-Whitney *U*检验进行比较。分类资料以频率（％）表示，采用*χ*²检验或Fisher精确概率法进行比较，利用Logistic回归模型进行多因素分析。采用Kaplan-Meier法描绘生存曲线，Log-rank检验比较生存差异，采用Cox回归模型进行多因素分析评估影响预后的独立因素，以*P*<0.05为差异有统计学意义。用GraphPad 10.5绘制统计图及生存曲线。

## 结果

1. 一般资料：DIC组患者中，老年患者（≥60岁）共13例（11.9％），男性患者共59例（54.1％），ELN风险分层低风险25例（22.9％），中风险44例（40.4％），高风险40例（36.7％）；非DIC组中，老年患者（≥60岁）共36例（12.1％），男性患者共155例（52.0％），ELN风险分层低风险122例（40.9％），中风险45例（15.1％），高风险131例（44.0％）。两组患者在年龄及性别方面差异无统计学意义（均*P*>0.05），DIC组中ELN分层为中风险组的患者比例更高（*P*<0.001）。两组FAB分型分布详见表1。

**表1:** 两组急性髓系白血病患者基本特征与接受治疗情况

临床特征	DIC组（109例）	非DIC组（298例）	*z*/*χ*²值	*P*值
年龄［例（％）］			0.002	0.966
≥60岁	13（11.9）	36（12.1）		
<60岁	96（88.1）	262（87.9）		
性别［例（％）］			0.143	0.750
男	59（54.1）	155（52.0）		
女	50（45.9）	143（48.0）		
骨髓原始细胞［％，*M*（范围）］	76.5（11.9～97.0）	55.0（6.0～98.5）	−5.467	<0.001
PT［s，*M*（范围）］	16.00（12.8～53.4）	14.25（10.80～18.90）	−8.993	<0.001
APTT［s，*M*（范围）］	39.30（22.10～68.20）	37.70（23.70～90.00）	−2.914	0.004
FIB［g/L，*M*（范围）］	2.72（0.34～7.70）	3.77（1.69～9.04）	−6.760	<0.001
D-二聚体［mg/L，*M*（范围）］	11.38（0.40～82.56）	0.75（0.05～20.00）	−14.599	<0.001
WBC［×10^9^/L，*M*（范围）］	40.7（0.4～383.8）	13.6（0.4～362.2）	−5.113	<0.001
HGB［g/L，*M*（范围）］	81.0（34.0～154.0）	82.0（27.0～161.0）	−0.103	0.671
PLT［例（％）］			10.107	0.001
≥50×10^9^/L	6（5.5）	54（18.1）		
<50×10^9^/L	103（94.5）	244（81.9）		
LDH［U/L，*M*（范围）］	600.2（148.4～7336.3）	333.75（107.60～9311.6）	−6.673	<0.001
ALT［U/L，*M*（范围））］	27.0（5.7～687.3）	16.3（4.1～167.3）	−3.982	<0.001
AST［U/L，*M*（范围）］	26.6（8.8～1703.4）	17.8（7.4～160.5）	−5.898	<0.001
TB［µmol/L，*M*（范围）］	10.1（3.9～146.0）	10.7（4.1～48.1）	−1.357	0.175
Cr［µmol/L，*M*（范围）］	61.6（32.8～196.0）	59.9（26.0～157.9）	−1.260	0.208
ELN2022分级［例（％）］			31.463	<0.001
低风险	25（22.9）	122（40.9）		
中风险	44（40.4）	45（15.1）		
高风险	40（36.7）	131（44.0）		
FAB分型［例（％）］^a^			20.897	0.001
M_0_	2（1.8）	1（0.3）		
M_1_	15（13.8）	19（6.4）		
M_2_	25（22.9）	101（33.9）		
M_4_	11（10.1）	53（17.8）		
M_5_	33（30.3）	46（15.4）		
M_6_	0（0）	2（0.7）		
化疗方案［例（％）］^b^			0.759	0.384
传统化疗方案	81（76.4）	238（80.4）		
包含维奈克拉的方案	25（23.6）	58（19.6）		
是否接受移植［例（％）］^b^			0.113	0.737
是	65（61.3）	176（59.5）		
否	41（38.7）	120（40.5）		

**注** DIC：弥散性血管内凝血；PT：凝血酶原时间；APTT：活化部分凝血活酶时间；FIB：纤维蛋白原；LDH：乳酸脱氢酶；ALT：丙氨酸转氨酶；AST：天冬氨酸转氨酶；TB：总胆红素；Cr：肌酐；ELN：欧洲白血病协作网。^a^DIC组有23例（21.1％）不能分型，非DIC组有76例（25.5％）不能分型；^b^共计5例患者因早期死亡未接受治疗

DIC组患者初诊时的白细胞计数水平、LDH、ALT、AST显著高于非DIC组（均*P*<0.001），DIC组患者的PT、APTT、D-二聚体水平均高于非DIC组（均*P*<0.05），FIB水平显著低于非DIC组（*P*<0.001）。骨髓原始细胞百分比较高（*P*<0.001），DIC组患者的血小板计数<50×10⁹/L占比更高（*P*＝0.005），两组间血红蛋白浓度、总胆红素和肌酐差异无统计学意义（*P*>0.05）。

治疗方面，407例患者中，除5例早期死亡患者外，其余402例患者均行诱导化疗，DIC组中有65例（65/106，61.3％）接受造血干细胞移植，非DIC组中有176例（176/296，59.5％）接受造血干细胞移植。两组患者在化疗方案及是否接受移植方面差异无统计学意义（均*P*>0.05）（表1）。

2. 细胞遗传学和分子生物学特点：根据ELN2022评分进行染色体核型分组，DIC组中低风险10例（9.2％）、中风险84例（77.1％）、高风险15例（13.8％），非DIC组中低风险59例（19.8％）、中风险182例（61.1％）、高风险57例（19.1％），组间差异具有统计学意义（*χ*²＝9.735，*P*＝0.008）。比较42种AML相关基因突变，DIC组NPM1（38.5％对15.8％，*P*<0.001）、FLT3-ITD（39.4％对19.2％，*P*<0.001）、SETD2（6.4％对2.0％，*P*＝0.025）基因突变比例均高于非DIC组。其余39种基因突变的发生频率在两组间差异无统计学意义（图1）。

**图1:**
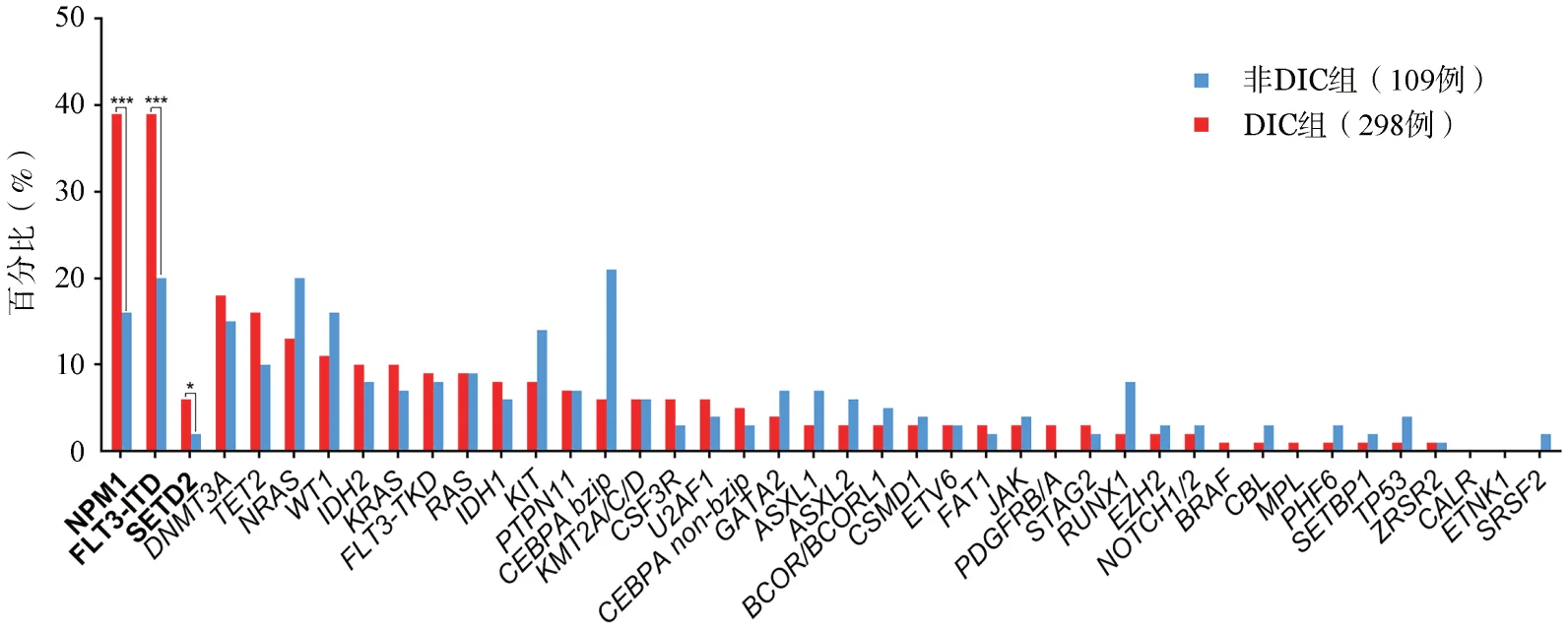
弥散性血管内凝血（DIC）组和非DIC组急性髓系白血病患者基因突变比较（***P<0.01，*P<0.05）

3. DIC危险因素Logistic多因素回归分析：将具有统计学差异的变量及临床重要变量年龄纳入Logistic回归模型，进行多因素分析，结果显示，原始细胞比例高、白细胞计数增高、乳酸脱氢酶高、NPM1突变是初诊非M_3_型AML发生DIC的独立危险因素（表2）。

**表2:** 急性髓系白血病合并DIC的危险因素多因素分析

变量	多因素分析	
	*OR*（95％ *CI*）	*P*值
年龄（≥60岁/<60岁）	1.104（0.524～2.325）	0.795
原始细胞比例（≥70％ /<70％）	1.872（1.121～3.125）	0.017
WBC（≥40×10^9^/L /<40×10^9^/L）	1.950（1.138～3.342）	0.015
LDH（≥1000 U/L /<1000 U/L）	2.746（1.389～5.428）	0.004
ALT（≥80 U/L /<80 U/L）	2.570（0.810～8.147）	0.109
AST（≥80 U/L /<80 U/L）	0.975（0.241～3.940）	0.971
NPM1突变	2.285（1.244～4.196）	0.008
FLT3-ITD突变	1.522（0.866～2.677）	0.144
SETD2突变	2.320（0.655～8.224）	0.192
染色体分型		
中风险/低风险	1.471（0.718～3.015）	0.291
中风险/高风险	0.900（0.341～2.372）	0.831

**注** DIC：弥散性血管内凝血；LDH：乳酸脱氢酶；ALT：丙氨酸转氨酶；AST：天冬氨酸转氨酶

4. 早期出血事件评价：407例患者中，共有153例（37.5％）患者在确诊至初次诱导化疗60 d内发生不同程度的出血事件，25例（6.1％）患者发生重度出血。在DIC组中，16例（14.7％）患者发生重度出血事件，而非DIC组，仅9例（3.0％）患者发生早期重度出血事件，DIC组患者的早期重度出血事件发生率显著高于非DIC组（*χ*²＝18.817，*P*<0.001）。

5. 治疗情况及生存分析：在接受化疗的402例患者中，DIC组有75例（75/106，70.8％）患者在初次诱导化疗后达到CR，非DIC组共237例（237/296，80.1％）患者达到CR，合并DIC的患者初次接受化疗的缓解率显著降低（*χ*²＝3.896，*P*＝0.048）。其中有241例（59.9％）患者接受了造血干细胞移植，生存分析显示接受造血干细胞移植的患者的生存明显优于未接受移植的患者（图2A）。

**图2:**
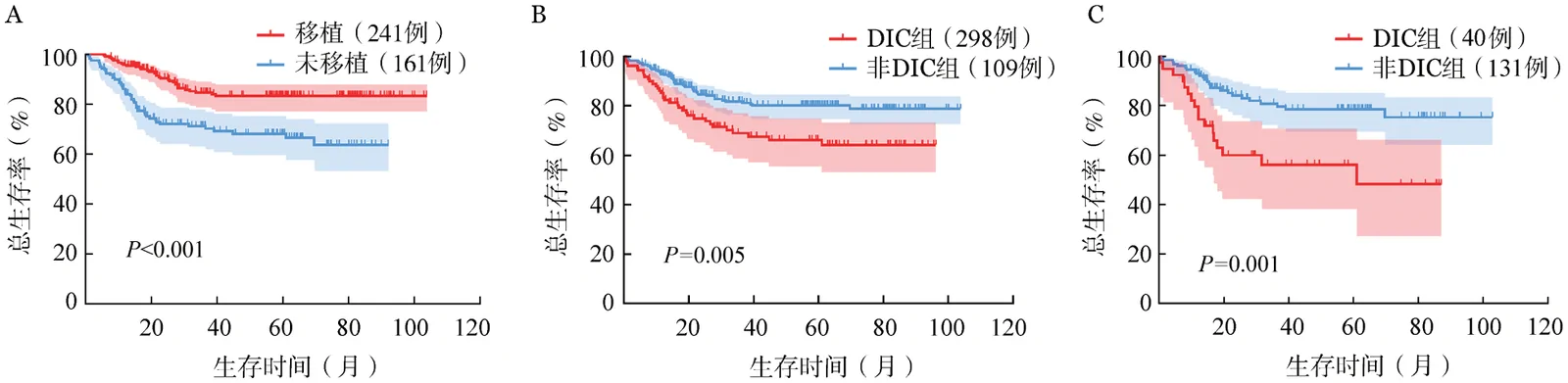
是否接受移植对急性髓系白血病（AML）患者生存的影响（A）及在总人群（B）和ELN2022高风险人群（C）中DIC组和非DIC组总生存比较

截至随访终点，86例患者死亡，其中DIC组34例，非DIC组52例，DIC组的生存期整体短于非DIC组（*HR*＝1.850，95％*CI*：1.200～2.851，*P*＝0.005）（图2B）。根据ELN2022分级进行亚组分析，分别比较DIC在不同预后分级人群中的影响，结果显示，在低、中风险人群中是否发生DIC对预后无显著影响（*P*>0.05）。而在高风险人群中，非DIC组的中位OS未达到，DIC组的中位生存期仅为61个月，DIC组的生存显著劣于非DIC组（*HR*＝2.655，95％*CI*：1.431～4.925，*P*＝0.001）（图2C）。

生存的单因素分析结果提示年龄、是否合并DIC、早期出血事件的发生、ELN预后分级是影响初诊非M_3_型AML患者OS的重要因素（*P*<0.05）（表3）。将上述单因素分析具有统计学差异的预后影响因素纳入Cox回归模型进行多因素分析，结果表明患者高龄（≥60岁）、合并DIC以及入院后早期发生重度出血事件为影响AML患者OS的独立危险因素（表3）。

**表3:** 影响急性髓系白血病患者总生存的单因素及多因素分析

变量	*HR*（95％ *CI*）	*P*值
单因素分析		
年龄（≥60岁/<60岁）	3.628（2.261～5.819）	<0.001
DIC（是/否）	1.850（1.200～2.851）	0.005
原始细胞比例（％）≥70 /<70	1.016（0.656～1.574）	0.942
WBC（≥40×10^9^/L /<40×10^9^/L）	1.390（0.898～2.153）	0.140
HGB（≥100 g/L /<100 g/L）	1.139（0.719～1.805）	0.580
LDH（≥1 000 U/L /<1 000 U/L）	1.571（0.934～2.641）	0.089
ALT（≥80 U/L /<80 U/L）	1.404（0.648～3.042）	0.389
AST（≥80 U/L /<80 U/L）	1.105（0.405～3.014）	0.846
Cr（≥120 µmol/L /<120 µmol/L）	1.414（0.348～5.750）	0.628
TB（≥30 µmol/L /<30 µmol/L）	2.145（0.869～5.295）	0.098
化疗方案（传统/包含VEN）	1.554（0.936～2.580）	0.088
首次疗程CR（是/否）	0.782（0.482～1.269）	0.320
早期大出血（是/否）	3.462（1.952～6.138）	<0.001
ELN2022预后分级		
低风险/高风险	0.527（0.312～0.889）	0.016
中风险/高风险	0.966（0.581～1.606）	0.894
多因素分析		
年龄（≥60岁/<60岁）	3.696（2.277～5.999）	<0.001
DIC（是/否）	1.651（1.017～2.683）	0.043
早期大出血（是/否）	2.849（1.542～5.261）	0.001

**注** DIC：弥散性血管内凝血；LDH：乳酸脱氢酶；ALT：丙氨酸转氨酶；AST：天冬氨酸转氨酶；TB：总胆红素；Cr：肌酐；ELN：欧洲白血病协作网；CR：完全缓解

## 讨论

既往关于DIC的研究多集中于APL[3]，非M_3_型AML相关DIC的报道相对有限。有报道AML患者初诊时WBC>100×10^9^/L时常合并DIC，并且有更高的早期死亡风险以及更差的远期预后[13]。本研究结果显示，初诊时合并高白细胞计数、骨髓原始细胞比例高及乳酸脱氢酶高是DIC发生的独立危险因素。既往研究指出诊断早期外周血白细胞计数升高及血乳酸脱氢酶升高与APL的出血事件密切相关[14]，本研究结果提示骨髓浸润程度、外周血白血病细胞负荷及代谢活性同样在AML患者DIC的发生中发挥重要作用。Hisada等[4]亦强调AML的凝血异常与高白细胞负荷及骨髓原始细胞比例升高密切相关，与本研究的结果一致。

本研究发现NPM1突变频率在DIC组显著升高，是非M_3_型AML患者发生DIC的独立危险因素，与既往研究相符合[15]–[16]。已有研究表明急性单核细胞白血病在年轻人中与DIC相关[17]，并且急性单核细胞白血病的患者常伴随NPM1突变[18]。先前研究表明，NPM1突变的AML患者的外周血白细胞计数与骨髓原始细胞比例显著升高[19]，与本研究结论一致，提示NPM1突变背景下的肿瘤负荷与凝血风险的关系值得进一步研究。在本研究中，DIC组的FLT3-ITD突变频率显著升高，已有研究表明合并高白细胞血症的AML患者中FLT3-ITD突变频率显著增高[19]，综上说明在NPM1突变以及FLT3-ITD突变的患者中有着更高的肿瘤负荷的倾向。Guo等[16]认为，NPM1和FLT3-ITD突变是非M_3_型AML患者并发DIC的独立危险因素，我们的研究结果显示NPM1突变是发生DIC的独立危险因素，提示NPM1突变在DIC发生发展中的重要作用，在多因素分析中，FLT3-ITD突变并未显示对于DIC的独立预测作用，其在凝血异常AML的机制中的作用需要进一步研究。有专家认为在DIC的治疗方面仍应该以治疗原发病为主，是否应早期启动抗凝治疗仍存在争议[20]，但对于AML的高危分子亚型，探索靶向药物联合抗凝治疗的可行性，有望改善DIC患者的预后。SETD2作为H3K36me3甲基转移酶，报道指出SETD2突变可使部分AML呈现出与APL相似的表型特征[21]。在我们的队列中，SETD2突变的发生频率仅为3.2％（13/407），关于SETD2突变对AML患者发生DIC的影响，需要更深入地研究。本研究发现，DIC组中ELN2022中风险患者比例高于非DIC组，但既往尚无文献证实ELN 2022评分可用于预测AML患者DIC发生风险，这一现象可能与NPM1和FLT3-ITD突变富集有关。染色体核型分析亦显示中风险核型组DIC发生率较高，但在多因素分析中未表现出独立作用，相关机制仍有待进一步研究。

伴随DIC的非M_3_型AML患者合并其他系统并发症的比例增加，并且AML患者诊断时合并DIC能够预测早期死亡[6],[22]，非M_3_型AML患者诊断时的DIC评分可预测化疗期间静脉或动脉血栓风险[23]。有研究提及重度出血事件的发生与患者更差的生存预后有关[24]，本研究发现合并DIC的患者早期大出血事件的发生率显著升高，提示早期动态监测血常规及凝血功能，预防出血尤为重要，此外，DIC组首次接受诱导治疗的缓解率低于未合并DIC的患者，表明DIC与难治性急性白血病之间可能存在一定的关联。生存分析结果显示，合并DIC的非M_3_型AML患者OS率显著降低，预后明显不良。尤其在ELN高危分层的患者中，初诊时伴DIC对OS期产生显著的不利影响。多因素分析中，在AML患者中高龄、合并DIC及早期发生重度出血事件是影响OS期的独立危险因素。临床上，对于初诊合并高白细胞计数，尤其伴NPM1突变、乳酸脱氢酶升高及血小板减少的患者，应高度警惕DIC发生，并结合DIC评分及动态监测尽早干预，以减少重度出血并改善预后。

本研究存在一定的局限性。本研究为单中心的回顾性分析，病例来源相对有限，可能存在选择偏倚；仅纳入了初诊或特定时间点的DIC实验室指标，缺乏对治疗过程中凝血动态变化的连续监测。本研究中，合并DIC的患者占总人群的26.8％，略高于既往文献报道的发生率[5]，可能与未排除感染等变量有关。期待未来多中心前瞻性研究验证NPM1等分子标志物对非M_3_型AML发生DIC的预测价值，结合基因表达水平、蛋白质变化等方面的分析，可以深入阐明其在DIC发生中的作用机制。
